# Super rapid learning of new attentional sets

**DOI:** 10.3758/s13423-025-02728-z

**Published:** 2025-07-07

**Authors:** Seth A. Marx, Sisi Wang, Geoffrey F. Woodman

**Affiliations:** 1 Department of Psychology, Vanderbilt Vision Research Center, Vanderbilt University, PMB 407817, 2301 Vanderbilt Place, Nashville, TN USA; 2 Institute for Brain and Behavior Amsterdam, Department of Experimental and Applied Psychology, Vrije Universiteit Amsterdam, Amsterdam, The Netherlands

**Keywords:** Attentional control, Visual long-term memory, Attentional guidance, Cognitive control

## Abstract

Humans guide attention to different targets as they navigate (e.g., going from a school zone to a construction zone while driving). To understand how observers shift focus between different sets of targets when the context shifts, we had observers perform a new visual search paradigm where target shapes are presented in multiple possible colors with distinct probabilities (e.g., 33%, 26%, 19%, 12%, and 10% baseline colors), such that some target colors are more task-relevant than others. Participants learned to prioritize the target colors that appeared with the target shape most often. We then scrambled the color-probability mapping to assess how quickly people could relearn a new set of target probabilities. Participants relearned these new colors significantly faster than their first set of colors, especially when there was less conflict between the new and old attentional sets. Our findings suggest that memory-guided attention is rapidly and continuously relearning to update task-relevant probabilities.

## Introduction

In our everyday lives, we guide our attention to different targets in ever-changing environments. For example, we may look for ripe fruit at a farmer’s market before jumping back into our car during rush hour, requiring us to attend to trucks, pedestrians, and other critical targets. As we shift between these task contexts, our attentional system needs to change the set of possible targets for which it looks (i.e., looking for the reddest apple versus looking for the red of stoplights). However, cognitive scientists have rarely studied attention in these dynamic settings. When we have, experiments show that it takes a significant amount of time for participants to acquire an association between a single color target when it has been paired with task-relevant target shapes (e.g., Conn et al., 2020; Cosman & Vecera, 2011). Our goal here was to understand how people can reconfigure their attentional systems to select a different slate of possible targets as they shift between different contexts that require them to look for different things, just as people commonly do driving vehicles.

**Fig. 1:**
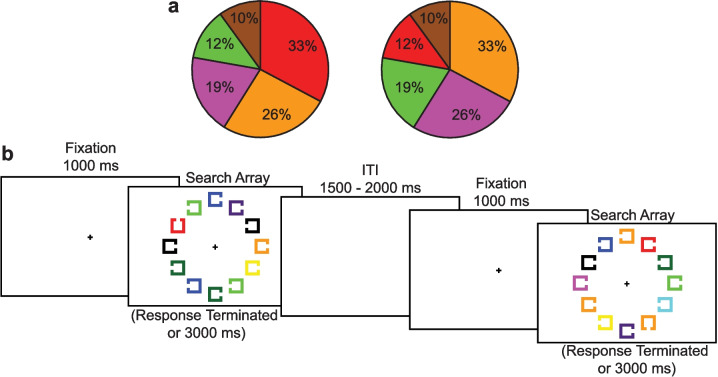
Example of the stimuli and procedure used in this study. (**a**) The pie chart shows an example of a color-probability distribution for an example participant, that then shifts to a different color-probability distribution in the second half of the experiment, shown with the right pie chart. (**b**) An illustration of the sequence of stimuli presented during two consecutive example trials

To determine whether multiple, incompatible attentional sets interfere with one another, we used a newly developed probabilistic visual search task (Wang et al., 2023; Wang & Woodman, 2024). The participants completed a visual search for shape-defined targets that required them to identify whether each array contained a target shape that had a gap on its top or its bottom (see Fig. 1a). The key manipulation involved presenting the target shape in different colors mapped to four unique probabilities (i.e., 33%, 26%, 19%, 12%) and a set of baseline colors for comparison (10% of the trials distributed across the remaining pool of colors). After searching for the shape targets through one set of color-probability mappings, we scrambled the colors, without informing the participants of this change, into a new attentional set of the same colors appearing as targets in different probabilities. We focus on how quickly they could learn this new, scrambled set of colors in this study to assess whether attentional control settings are being dynamically updated. In Experiment 1, we conducted an in-laboratory experiment and directly manipulated whether the two attentional sets were more or less similar. In Experiment 2, we shortened the experiment for the online setting, using two blocks of 400 trials to observe the initial learning and the subsequent relearning. In Experiment 3, we introduced another equally spaced attentional set change during the experiment to determine if interference might compound across attentional sets as people acquire multiple conflicting sets of colors across time.

If learning to attend to an array of possible target features is like learning other information, then we should see that the old attentional set initially causes interference (i.e., an increase in response time (RT) and a decrease in accuracy) before learning the new attentional set returns performance to levels similar to those observed before the shift in context, and this should take as long or longer than learning the first set of target information (Reinhart & Woodman, 2015; Reinhart et al., 2019; Woodman et al., 2013). On the other hand, perhaps learning to attentionally forage is an ongoing process, always updated with new information from the environment and accumulating what it learns in long-term memory (Hutchinson & Turk-Browne, 2012; Servant et al., 2018; Woodman et al., 2013). By extracting statistics continuously, human visual attention can retune itself to the biases inherent in the environment (Awh et al., 2012; Reinhart & Woodman, 2015; Woodman & Chun, 2006; Zhao et al., 2013). If this is how the human attentional system works, then across time, the system should learn faster as it rules out possible predictive power from pure-noise features both in the room and on the experimental screen (e.g., such as the lighting in the experiment room or the hue of the background color). In the world outside the laboratory, shifts of context are so pervasive that our minds may be configured such that attentional sets cause minimal mutual interference by essentially being continuously updated, unlike learning memoranda that must be explicitly retrieved from memory in which competitive interference is ubiquitous (Endress & Szabó, 2017; Underwood, 1957). As shown below, participants’ performance supported the predictions of faster relearning of a new attentional set compared to the initial learning rate, supporting the idea that feature-based attentional sets appear to be continuously extracted from the environment to efficiently guide attention in dynamic environments.

## Methods

### Participants

For Experiment 1, we recruited 22 participants from Vanderbilt University, each compensated using course credit or $15 per hour for their time. We used behavioral effect sizes derived from a previous event-related potential study to estimate sample sizes with G*Power that would result in power of 0.8 (Wang et al., 2023; Faul et al., 2007); we estimated that 16 participants should be sufficient. For Experiment 2, 140 participants were recruited from Prolific and paid a mean of US$10.49 per hour. For Experiment 3, 240 participants were recruited from Prolific and 26 students were recruited from Vanderbilt University. Prolific participants were paid a mean of US$10.84 per hour, and Vanderbilt students were compensated with course credit. Online participant data sets were used in the analyses if accuracy in each of the learning phases was above 60% correct, there was at least one correct response for each probability in every block, and no more than 15 unanswered trials in any session of Experiment 2 and ten unanswered trials in any session of Experiment 3. After removing participants using these criteria, we were left with 132 participants in Experiment 2 and 205 participants in Experiment 3 for final analyses.

### Stimuli

Figure 1a shows an example color distribution from Experiment 1, and Fig. 1b illustrates an example of the stimulus sequence. The stimuli were presented on a white background (254 cd/m^2^). Each trial began with a black fixation cross presented in the center of the screen for 1,000 ms (0.51 cd/m^2^, 0.4° X 0.4° of visual angle, assuming a natural viewing distance of 75 cm from the monitor). Next, we presented a search array containing one target square (with an opening on the top or the bottom of the square) and 11 distractor squares (with openings on the left or the right). This search array was presented until participants made a response on each trial. The squares were unfilled shapes 0.7° × 0.7° visual angle, and line thickness of 0.1° visual angle, with an opening that was a gap 0.5° visual angle wide on one of the four edges. Each square was randomly placed at one of 12 possible locations in a ring around fixation (from 0° to 330° with a step size of 30° of polar angle) with an eccentricity of 4.4° visual angle. After the search array terminated with the participant’s response, or after a maximum of 3,000 ms, a blank screen appeared with a variable inter-trial interval ranging from 1,000 ms to 1,500 ms (randomly sampled from a rectangular distribution).

For Experiment 1, the target shape similarly appeared in one of eight highly distinguishable colors: red (x = 0.616, y = 0.337, in CIE color space, 14 cd/m2), green (x = 0.284, y = 0.958, 44 cd/m2), magenta (x = 0.295, y = 0.153, 19.2 cd/m2), yellow (x = 0.407, y = 0.504, 54.4 cd/m2), blue (x = 0.146, y = 0.720, 6.5 cd/m2), cyan (x = 0.432, y = 0.405, 16.1 cd/m2), black (> 0.01 cd/m2), and orange (x = 0.552, y = 0.397, 24.2 cd/m2). Targets appeared in each color with unique probabilities, and we plotted and analyzed performance separately for the top four (i.e., 33%, 26%, 19%, and 12%), collapsing across the four baseline colors composing the remaining 10% of all trials (with the different colors having targets in their color 5%, 2%, 2%, and 1% of trials). For Experiment 2, the colors of the squares were chosen from a pool of ten highly discriminable colors: red (x = 656 y = 325, using the 1931 CIE coordinates), green (x = 336 y = 592), magenta (x = 430 y = 592), yellow (x = 468 y = 482), blue (x = 165 y = 71), cyan (x = 272 y = 395), black (x = 323 y = 275), orange (x = 553 y = 413), dark green (x = 327 y = 598), and purple (x = 303 y = 143).

For the in-person experiment, to better characterize the flexibility of attentional set learning, we intentionally manipulated the degree of overlap between the new learning phase and the preceding learning phase in the laboratory. Specifically, we collected data from two groups of participants. One group completed the small-shift version (the 33% and 26% colors in Phase 1 switched to 19% and 12% in Phase 2, the 19% and 12% colors in Phase 1 switched to 33% and 26% in Phase 2, and the baseline colors in Phase 1 were still used as baseline colors in Phase 2 but shuffled their probabilities), and another group completed the large-shift version (the 33%, 26%, 19%, and 12% colors in Phase 1 switched to the baseline-probability colors in Phase 2, and the baseline colors in Phase 1 switched to 33%, 26%, 19%, and 12% colors in Phase 2). The key difference between the two groups is that for the small-shift group the probability change for each color between the two learning phases is relatively small, while for the large-shift group the probability change for each color between the two learning phases is more dramatic. With this design, we infer the degree to which new learning suffers interference from previously learned regularities.

In Experiments 2 and 3, the target shape appeared in one of ten highly distinguishable colors, with four of them paired with high probabilities (i.e., 33%, 26%, 19%, and 12%), and the remaining six colors as baseline composing the remaining 10% of all trials (with the different colors having targets in their color 5%, 1%, 1%, 1%, 1%, and 1% of trials). For each new learning phase in the online experiment, the colors were randomly mapped to new probabilities in the distribution set (i.e., the color-probability matches are selected independently in both learning phases).

### Procedure

Experiment 1 was run in the laboratory using MATLAB and the Psychophysics toolbox (Brainard, 1997), with observers at 75 cm viewing distance. Experiments 2 and 3 were web-hosted online using Cognition.run and programmed using the jsPsych 7.3.1 library (De LeeuW, 2015). Participants were instructed to respond as fast and as accurately as possible. Participants used the F key and the J key on a keyboard to report whether the target had its gap on the top or the bottom. The button mapping was counterbalanced across participants.

The target colors, target locations, and target shape (gap up or down) were randomized. Trials with targets with a gap up versus a gap down were equally likely across the experiment (i.e., each 50%). For each search array, colors of distractors were randomly chosen from the color pool with no more than two distractors appearing in the same color. The probability-matching colors were randomized across participants to prevent potential confounds from inherent visual preferences for different colors that are shared across people (e.g., preferring to search for red or green due to these colors in traffic lights).

For Experiment 1, each participant completed two 1,000-trial phases. The data were collected in person in the laboratory to achieve more stable statistical estimates and better control of the experimental environment. In Experiment 2, each participant completed two 400-trial phases. In Experiment 3, each participant completed three 200-trial phases. Due to the online nature of Experiments 2 and 3, we had fewer trials per participant and collected more participants than in Experiment 1. We note that we also ran another online experiment similar to Experiment 2 with 200 trials in each of two phases, rather than 400 trials in each phase. That experiment showed the same pattern of faster relearning of the new color set but failed to achieve a significant three-way interaction due to noise in the online sample. This appears to have been due to an unacceptably large number of participants having runs of trials in which they did not make any button-press responses.

The participants first performed a practice block of 20 trials with feedback. The practice trials presented two target objects in each possible color so that the probability distribution across colors was rectangular. The goal of the task was so that participants will learn the general task, with research suggesting that 20 trials of general task learning is sufficient to move performance past the initial acquisition stage and into the asymptote of the general learning curve (Logan, 2002).

## Results

**Fig. 2:**
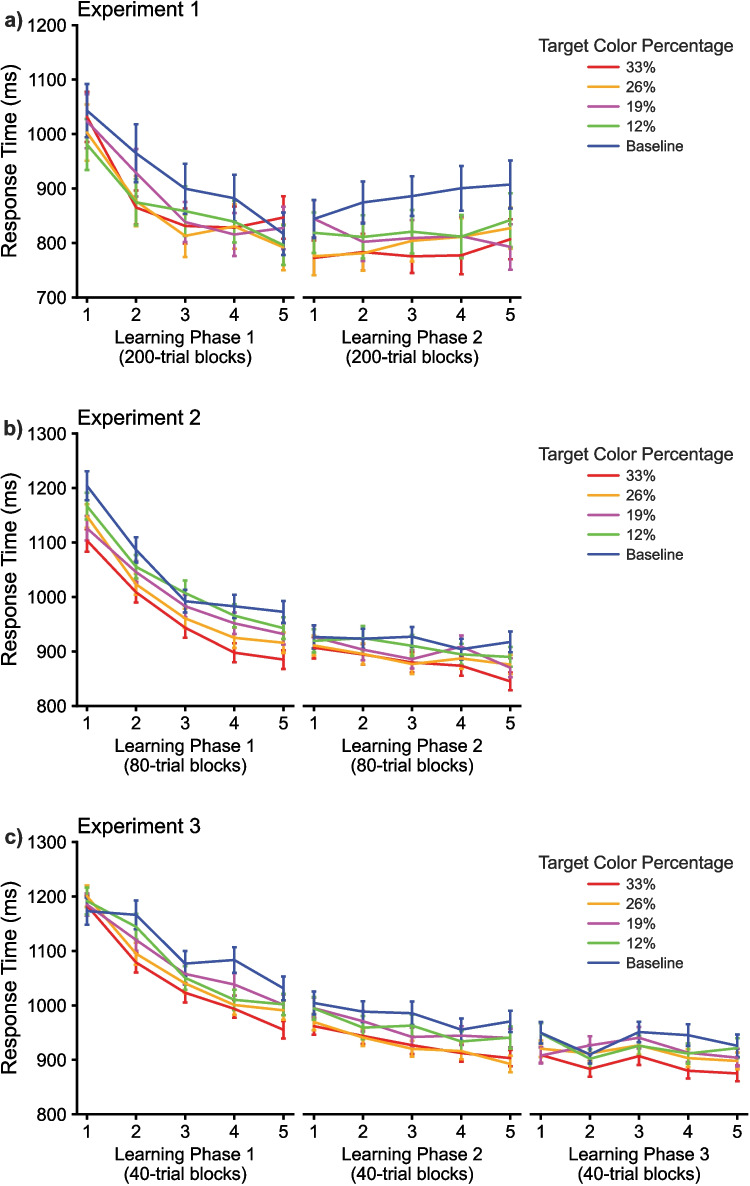
The response time (RT) results with accuracy shown in Fig. 3. (**a**) The plot shows the mean RTs across probability colors from learning phases 1 and 2 in Experiment 1. (**b**) The RT results from Experiment 2, the online replication and extension of Experiment 1. **(c**) The RT results from Experiment 3, the online extension of Experiments 1 and 2

**Fig. 3:**
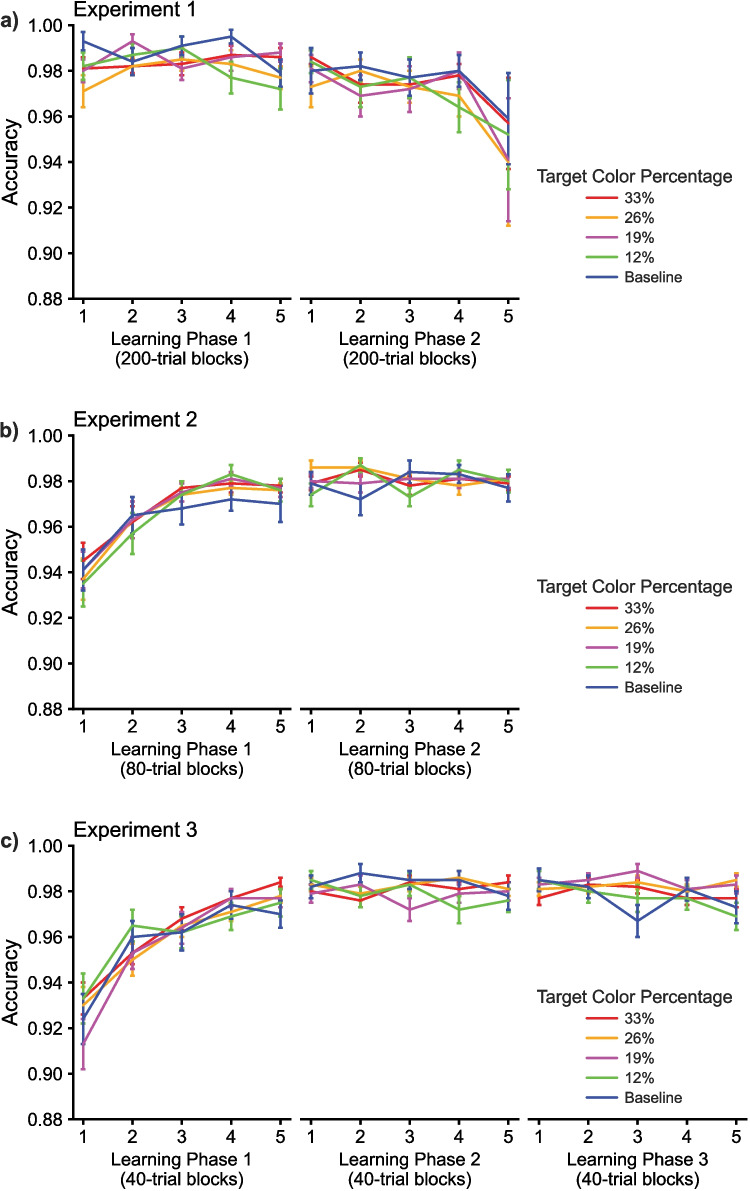
The accuracy data from Experiments 1, 2, and 3, shown in panels **a**,** b**, and **c**, respectively. The results demonstrate that accuracy was high and stable, or improved across blocks, similar to response times shown in Fig. 2, with no evidence of a speed-accuracy tradeoff

The findings from Experiment 1 are shown in Figs. 2a and 3a. As shown in Fig. 2a**,** participants responded faster to targets appearing in colors with higher target probabilities compared to lower probability colors. A three-way repeated-measures ANOVA with within-participant factors of Block (1–5), Color Probability (33%, 26%, 19%, 12%, and Baseline), and Learning Phase (Phase 1 vs. Phase 2) was applied to participants’ RTs. The analysis showed a significant main effect of Block (*F*(4,84) = 11.695, *p* < 0.001, η^2^ = 0.358), Color Probability (*F*(4,84) = 8.202, *p* < 0.001, η^2^ = 0.281), Learning Phase (*F*(1,21) = 16.163, *p* = 0.001, η^2^ = 0.435), and significant interactions between Block x Color Probability (*F*(16,336) = 2.218, *p* = 0.005, η^2^ = 0.096), between Block x Learning Phase (*F*(4,84) = 37.573, *p* < 0.001, η^2^ = 0.641), and a three-way interaction between Block x Color Probability x Learning Phase (*F*(16,336) = 2.346, *p* = 0.003, η^2^ = 0.100).

A three-way repeated measures ANOVA using the same within-participant factors was applied to the accuracy data. The results showed no evidence of a speed-accuracy tradeoff due to the color-probability manipulation across learning phases. This analysis did not show a significant effect of Block (*F*(4,84) = 1.750, *p* = 0.147, η^2^ = 0.077) but did show a significant main effect of Color Probability (*F*(4,84) = 3.554, *p* = 0.010, η^2^ = 0.145) and marginal main effect of Learning Phase (*F*(1,21) = 3.992, *p* = 0.059, η^2^ = 0.160). The analysis did not yield any significant interactions (*p*s > 0.05).

The significant three-way interaction on RT in Experiment 1 is notable as it supports the conclusion that people learn faster across blocks. To decompose this interaction, we applied repeated-measures ANOVAs with the factor of Color Probability to each block separately to determine at which block the regularity learning was established and found that participants rapidly learned the regularity after the first 200-trial block in learning phase 1 (for the first block: *F*(4,84) = 1.403, *p* = 0.240, η^2^ = 0.063, and the later four blocks *p*s ≤ 0.058). Whereas they learned the regularity even at the first block in learning phase 2 (*p*s ≤ 0.014 across all five blocks). Again, these results support the idea that attentional sets are continuously relearned and updated across time. This allows the system to quickly adjust as the statistics of the environment shift.

**Fig. 4:**
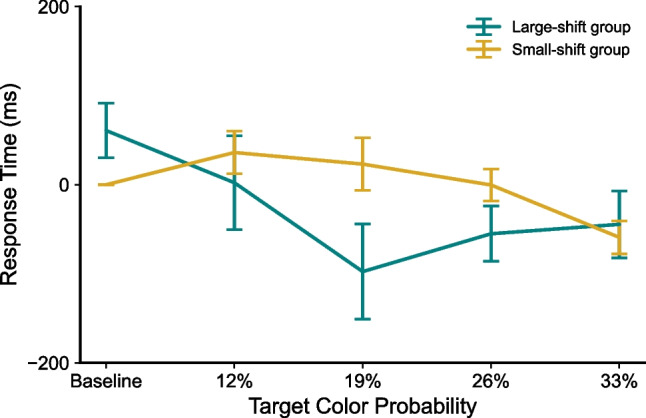
The difference in search response times (RTs) from both groups in Experiment 1, sorted as a function of the RT difference between RTs sorted by the first phase colors minus RTs sorted by the second phase colors. Lasting guidance from the old colors interfered more with performance in the second phase for the small-shift group

We next compared the learning pattern between two groups of participants from our laboratory experiment. Both groups showed rapid learning and relearning of the color-distributions regardless of the magnitude of the change (see Fig. 4). However, we also observed a stronger carry-over interference effect from the previous attentional set in the small-shift group than in the large-shift group, in which the color sets were more different. This pattern is verified by mixed repeated-measures ANOVAs with the between-participant factor of Group (large-shift group vs. small-shift group) and a within-participant factor of Target Color Probability (four unique probabilities and the collapsed baseline) on RTs across learning phases. First, the mixed ANOVA on RTs in learning phase 2 with the new target-color probability distribution revealed only a main effect of Target Color Probability (*F*(4,80) = 7.089, *p* < 0.001, η^2^ = 0.262), while no main effect of Group (*F*(1,20) = 3.040, *p* = 0.097, η^2^ = 0.132) or interaction between Group and Probability was found (*F*(4,80) = 1.394, *p* = 0.2444, η^2^ = 0.065), suggesting the successful updating of new regularity in both groups.

Here we analyzed the RT data from Phase 2 as a function of the target color probability from Phase 1 to assess whether the first color distribution that was learned was interfering with subsequent learning. If this was occurring, then the interference would be dependent on the size of the probability changes across color space. Figure 4 shows the large-shift group and small-shift group data separately, sorted by the RT difference between the old color-distribution in Phase 1 and the new color distribution in Phase 2. In a plot such as this, better learning of the new color set would result in a sigmoid function where frequent targets are speeded, and rare color target responses are slowed. Figure 4 shows better relearning for the large-shift group, evidenced by the slowing of baseline RTs and speeding of high-probability target colors, moving off from the zero RT axis. An ANOVA on the RTs from learning Phase 2 using the old target-color probability distribution (from the preceding learning phase) showed a significant interaction between Group and Probability (*F*(4,80) = 4.415, *p* = 0.003, η^2^ = 0.181) and a significant Group difference (*F*(1,20) = 5.445, *p* = 0.030, η^2^ = 0.214). The pre-planned comparisons revealed a significant Prior Probability effect in the small-shift group (*F*(4,36) = 7.703, *p* < 0.001, η^2^ = 0.461), not the large-shift group (*F*(4,44) = 1.410, *p* = 0.246, η^2^ = 0.114). These findings suggest that participants who had to relearn small probability changes had more trouble relearning than those who experienced larger changes in the distribution of targets across colors. These findings demonstrate greater interference between neighboring or even overlapping attentional sets, consistent with findings from task-switching paradigms (Koch et al., 2018).

The results from Experiment 2, shown in Figs. 2b and 3b, follow the same pattern of results as Experiment 1. Participants responded faster to targets appearing in colors with higher target probabilities compared to lower probability colors, as this effect took approximately 80 trials to emerge. This was statistically supported by separate omnibus ANOVAs with within-participant factors of Block (1–5), Color Probability (33%, 26%, 19%, 12%, and Baseline), and Learning Phase (Phase 1 vs. Phase 2) on participants’ RT and accuracy.

The repeated-measures ANOVA on RTs yielded significant main effects of Block (*F*(4,524) = 133.278, *p* < 0.001, η^2^ = 0.504), Color Probability (*F*(4,524) = 21.544, *p* < 0.001, η^2^ = 0.141), and Learning Phase (*F*(1,131) = 232.273, *p* < 0.001, η^2^ = 0.639). Interaction effects were obtained for Learning Phase x Block (*F*(5,524) = 67.320, *p* < 0.001, η^2^ = 0.339), Learning Phase x Color Probability (*F*(4,524) = 2.477, *p* = 0.043, η^2^ = 0.019), and a three-way interaction for Learning Phase x Block x Probability (*F*(16,2096) = 1.680, *p* = 0.044, η^2^ = 0.013).

The analysis of accuracy showed no evidence of a speed-accuracy tradeoff. A main effect was not shown for Color Probability (*F*(4,524) = 0.931, *p* = 0.446, η^2^ = 0.007). There were main effects shown for Block (*F*(4,524) = 15.305, *p* < 0.001, η^2^ = 0.105), due to fewer errors from block 1 to 5 in both phases, and Learning Phase (*F*(1,131) = 27.732, *p* < 0.001, η^2^ = 0.175). The only interaction effect present was for Learning Phase x Block (*F*(4,524) = 11.207, *p* < 0.001, η^2^ = 0.079), with the three-way interaction not present (*F*(16,2096) = 1.153, *p* = 0.299, η^2^ = 0.009).

The most critical observation to test the prediction that relearning should be faster than initial learning is what happens in the second phase of the experiment when the colors are scrambled. What we observed was high-speed relearning of the color mappings such that the interactions obtained were for Block x Learning Phase due to the rapid drop in RTs from Block 1 to 2 in Phase 1 that was absent in Phase 2. That is, the drop in RTs in Phase 1 as participants learned was not observed in Phase 2, even though participants needed to learn a new color probability mapping that should be in conflict with what they had learned in the previous phase. Instead, we observed rapid relearning that was faster than initial learning. Critical for this claim, the RT ANOVA yielded a three-way effect of Phase x Block x Color Probability.

The rapid relearning indicates that the attentional system continues to relearn

attentional weights from the environment. But is this really continuous? Or does the attentional system become less responsive to environmental change across time as it uses the accumulated episodes to form an attentional set to harvest resources? To test this possibility in Experiment 3, we induced another color change after the second phase of the experiment, resulting in participants relearning twice and using three different color-target probability maps during the course of the experiment.

The results from Experiment 3, shown in Figs. 2c and 3c, follow the same pattern of results as Experiments 1 and 2. The repeated-measures ANOVA on RT showed significant main effects of Block (*F*(4,816) = 82.927, *p* < 0.001, η^2^ = 0.289), Color Probability (*F*(4,816) = 25.531, *p* < 0.001, η^2^ = 0.111), and Learning Phase (now with three levels; *F*(2,408) = 295.202, *p* < 0.001, η^2^ = 0.591). A marginal interaction effect was present for Block x Color Probability (*F*(16,3264) = 1.522, *p* = 0.083, η^2^ = 0.007), while a significant interaction was present for Learning Phase x Block (*F*(8,1632) = 32.988, *p* < 0.001, η^2^ = 0.139). The three-way interaction was also present on RT (*F*(32,6528) = 1.506, *p* = 0.034, η^2^ = 0.007).

Accuracy analyzed with a RM ANOVA did not show evidence of a speed-accuracy tradeoff. A main effect was not shown for Color Probability (*F*(4,816) = 0.639, *p* = 0.635, η^2^ = 0.003), but main effects were present for Learning Phase (*F*(2,408) = 37.998, *p* < 0.001, η^2^ = 0.157) and Block (*F*(4,816) = 15.957, *p* < 0.001, η^2^ = 0.073). A marginal interaction was present for Learning Phase x Color Probability (*F*(8,1632) = 1.880, *p* = 0.059, η^2^ = 0.009), and significant interactions were present for Learning Phase x Block (*F*(8,1632) = 21.063, *p* < 0.001, η^2^ = 0.094) and Block x Color Probability (*F*(16,3264) = 1.922, *p* = 0.015, η^2^ = 0.009). The three-way interaction was not significant (*F*(32,6528) = 0.914, *p* = 0.562, η^2^ = 0.005).

We obtained the same general pattern of RTs observed in Experiments 2 and 3 as we did in Experiment 1, although our in-laboratory participants appear to relearn even faster following the color shift. The other difference is that we see RTs converging in the last block of trials in the first phase. The participants were given feedback that they had one more block of trials before a long break (e.g., in which they could use the bathroom). We believe that this likely resulted in a spurious range effect in this block.

The accuracy data from our laboratory Experiment 1 differed from those of the online experiments. Primarily, this was due to accuracy starting off near ceiling in block 1 of Experiment 1 and decreasing across time, presumably due to fatigue. In contrast, accuracy slowly increased in Experiments 2 and 3 because our online participants needed more time to reach ceiling accuracy. The ANOVA on accuracy rates from Experiment 1 yielded a marginal main effect of Phase and significant main effect of Color Probability but no other main effects or interactions, df adjusted for non-sphericity using Greenhouse–Geisser.

## Discussion

Here we demonstrate that human participants can rapidly relearn attentional control settings. Additionally, the process by which this occurs appears to be continuous. When the task environment changed (i.e., colors randomly reassigned priority values without notifying the participants), participants rapidly relearned the new color priority mapping, seemingly benefitting from previous learning of attentional priorities. That is, in Experiment 1 we found that learning was actually significantly faster in the second phase of learning. In Experiment 2, we replicated these results with a larger online sample that performed fewer trials across shorter blocks. Finally, in Experiment 3 we examined whether this fast relearning continues throughout time. This involved inducing two changes of color probability such that participants learned three color sets. We saw that people continued to learn new color sets faster than they had learned the initial set.

Based on accounts of statistical learning and contextual cueing, participants are acquiring memories of associations between color and targethood that guide attention. We assume that the task set for the participants includes what response is to be made to each target shape and in which colors those target shapes will appear. However, it is possible that the task set includes information about features that can be reliably ignored. During the experimental session, it is possible that the participants are learning which colors appear more frequently as distractors (i.e., the orange power light on the monitor is never a target color, so I can always ignore it). Because some of these features are stable non-targets in the environment (e.g., the orange power light, the edge of the monitor, the experimenter’s face, the fixation point, etc.), observers can learn these and set their attentional priority to zero for these *never-targets*. Perhaps it is this process of learning never-targets in the task context that allows the visual system to become increasingly fast at reconfiguring the set of possible target features across the experiment. Subsequent experiments could test this idea by introducing novel distractors that are never targets into the arrays and verifying that they slow the acquisition of task-relevant sets of attentional control settings.

The present findings have important theoretical implications. Specifically, many of the prominent accounts of attention propose that attention is guided by representations that are held in working memory (Bundesen et al., 2005; Desimone & Duncan, 1995; Olivers et al., 2011; Wolfe & Horowitz, 2004). It is possible that this underlies why such memory representations do not show interference, as one of the primary functions that working memory is supposed to play is to prevent interference from long-term memory (Kane & Engle, 2002), and perhaps holding an attentional set active in working memory is more precise across time, and can include a rich mosaic of colors. However, it is also possible that our use of color sets to guide attention can be rapidly automatized. Under the instance theory of automatization (Logan, 2002), it is proposed that long-term memory representations become so potent as to control processing themselves, without the need for active maintenance in working memory. Although neuroscientific findings appear to make the working memory explanation for the present results unlikely (Wang et al., 2023), we believe that future work is needed that focuses on understanding the nature of the memory representations that can support such flexible and richly textured attentional sets.

## Data Availability

Datasets are available via the Open Science Framework (OSF) at: https://osf.io/jwhax/.

## Code availability

Available via the OSF: https://osf.io/jwhax/.
